# Do Promotions Make Consumers More Wasteful? The Effect of Price Promotion on Consumer Food Waste Behavior

**DOI:** 10.3390/bs16040495

**Published:** 2026-03-26

**Authors:** Yan Wang, Wei Xu, Emine Sarigöllü

**Affiliations:** 1 College of Economics and Management, Zhejiang A&F University, Hangzhou 311300, China; wangamelieyan@163.com; 2 School of Business and Management, Jilin University, Changchun 130012, China; 3 Desautels Faculty of Management, McGill University, Montreal, QC H3A 1G5, Canada; emine.sarigollu@mcgill.ca

**Keywords:** consumer food waste, price promotion, perceived resources, sustainable consumption, mental accounting

## Abstract

Consumer food waste is a major global challenge to sustainable development, generating massive carbon and water footprints, exacerbating food insecurity, and undermining the United Nations Sustainable Development Goals. While extensive research has documented individual and contextual drivers of consumer food waste, critical gaps remain in understanding how core marketing tools shape wasteful behavior, particularly the unintended post-purchase consequences of ubiquitous price promotions. Addressing this gap, we unpack the psychological mechanism underlying the classic social dilemma of promotions: short-term individual economic savings from discounts conflict with long-term collective ecological welfare. Across four rigorous studies, including a real-world field experiment in a university canteen, we establish a causal effect of price promotions on increased consumer food waste behavior. We further demonstrate that this effect is mediated by enhanced perceived resources: price promotions generate subjective feelings of windfall gains and resource abundance, which in turn increase consumers’ willingness to discard edible food. We identify two practical actionable boundary conditions that attenuate this pro-waste effect: the impact of price promotions on food waste is eliminated when consumers focus on money spent (rather than money saved) from the transaction, and when they perceive their spending as exceeding their psychological budget. Our findings advance the literature on price promotions and sustainable consumption by documenting a previously unrecognized hidden cost of promotional marketing, unpacking the micro-psychological foundations of the social dilemma in food waste decisions, and providing evidence-based, actionable implications for policymakers, food retailers, and food service operators to curb promotion-induced food waste.

## 1. Introduction

Food waste represents one of the most pressing global challenges to sustainable development, with approximately one-third of all food produced for human consumption lost or wasted annually worldwide (Schanes et al., 2018; United Nations, 2023). This massive inefficiency inflicts profound and irreversible harm across economic, social, and environmental dimensions: it causes trillions of dollars in direct annual economic losses, exacerbates global food insecurity and inequities in food access, and drives over 20% of global greenhouse gas emissions while depleting scarce freshwater and arable land resources (Gao, 2025; Stancu et al., 2016). Recognizing this urgency, the United Nations aims to half per capita global food waste at the retail and consumer levels by 2030 as a core target of Sustainable Development Goal (SDG) 12 (Responsible Consumption and Production) and explicitly identified consumer-level food waste reduction as the most actionable lever to achieve this goal (United Nations, 2023). Consumers are indeed the largest contributors to this crisis: consumer-level food waste accounts for roughly 57% of total food waste across the entire food supply chain, far exceeding the share of production, transportation, and retail links (Aschemann-Witzel et al., 2015; United Nations, 2023). In this research, we formally define consumer food waste behavior as consumers’ intentional or unintentional discarding of edible food that remains safe and nutritious for human consumption, including but not limited to unfinished restaurant/canteen plate waste, unconsumed food within its shelf life, edible produce with suboptimal appearance, and food products that do not meet taste expectations but are still fit for consumption (Gao, 2025; Schanes et al., 2018).

At its core, consumer food waste embodies a classic and pervasive social dilemma: individual consumption decisions driven by immediate self-interest (e.g., economic savings from promotions, convenience of discarding unsatisfactory food) collectively generate enormous long-term costs for collective ecological welfare and social sustainability (Van Doorn, 2016). The extant literature has extensively explored the drivers of this dilemma, which can be broadly categorized into two streams. The first stream focuses on intra-individual psychological factors, documenting that attitudes, subjective norms, perceived behavioral control (rooted in the theory of planned behavior), and anticipated emotions (e.g., guilt) are core predictors of consumers’ food waste decisions (Russell et al., 2017; Stefan et al., 2013). The second stream examines external contextual antecedents, ranging from micro-level factors such as social dining interactions and food sensory characteristics to macro-level factors including cultural norms and economic fluctuations (Gao, 2025; Qian et al., 2021). While these studies have advanced our understanding of food waste, they have largely overlooked the role of core marketing mix activities, which are the most pervasive and direct shapers of everyday consumer decisions, in influencing food waste behavior (Ilyuk, 2018; Mookerjee et al., 2021). Leading marketing scholars have repeatedly called for more systematic research into how marketing tools shape consumer food waste to bridge the critical gap between marketing practice and global sustainable consumption goals (Block et al., 2016; Van Doorn, 2016).

Emerging research at the intersection of marketing and food waste has begun to address this gap, documenting that consumers’ food waste behavior is influenced by perceived resource abundance (Porpino et al., 2016), plate size and material (Williamson et al., 2016), product ownership duration (Xie & Bagchi, 2024), scarcity mindset (Gao et al., 2024), and purchase channel differences (Ilyuk, 2018). However, this growing body of research has largely neglected one of the most ubiquitous, impactful, and strategically central elements of the marketing mix: price promotions. As a foundational tool for food retailers and brands worldwide, price promotions are designed to drive sales volume, shape consumer purchase decisions, and capture market share (Ailawadi et al., 2007; Chandon et al., 2000). Decades of marketing research have yielded rich insights into the effects of price promotions, from accelerating purchase timing and triggering impulse buying in pre-purchase stages to reducing perceived product efficacy in post-purchase stages (Bell et al., 1999; Heilman et al., 2002; Shiv et al., 2005). Despite this extensive literature, three critical and interconnected gaps remain unaddressed.

First, we know surprisingly little about the causal effect of price promotions on consumers’ post-purchase food waste behavior. The limited correlational research has speculated that promotion-induced stockpiling may lead to household food spoilage (Brizi & Biraglia, 2021; Lehberger et al., 2021), but these studies do not establish causal evidence, nor do they examine food waste beyond stockpiling-related spoilage, such as the discarding of edible food that fails to meet taste expectations, a major component of consumer food waste (Farr-Wharton et al., 2014). Second, there is no systematic research unpacking the psychological mechanism that links price promotions to consumer food waste. Without understanding the “why” behind this effect, we cannot explain how a marketing tool that delivers economic savings to consumers ends up increasing wasteful behavior that contradicts long-term sustainability. Third, no research has identified actionable boundary conditions that can mitigate the unintended wasteful consequences of price promotions, which is essential for translating theoretical insights into managerial and policy interventions.

These gaps leave three core research questions unanswered:

1. Do price promotions have a causal effect on increasing consumer food waste behavior?

2. What is the underlying psychological mechanism that explains the impact of price promotions on consumer food waste?

3. Under what conditions can the positive effect of price promotions on consumer food waste be attenuated?

The primary objective of this research is to address these gaps by developing and empirically testing a theoretical framework that bridges marketing strategy, sustainable consumption, and consumer decision psychology. Specifically, we aim to: (1) establish the causal effect of price promotions on consumer food waste behavior across controlled experimental and real-world field settings; (2) unpack the mediating psychological mechanism that drives this effect; (3) identify theoretically grounded and managerially actionable boundary conditions that weaken the promotion-induced increase in food waste; and (4) provide evidence-based insights for businesses and policymakers to align promotional marketing practices with sustainable consumption goals. This research delivers a mechanistic contribution to the interdisciplinary literature at the intersection of marketing, sustainability, and consumer psychology, addressing longstanding calls for more process-focused research on consumer food waste. We test our theoretical model across four complementary studies, combining controlled experiments and a real-world field experiment to provide convergent causal evidence for our predictions.

## 2. Literature Review and Hypotheses Development

### 2.1. Theoretical Anchoring

This study grounds its conceptual framework in two well-established theoretical perspectives: the Stimulus–Organism–Response (S-O-R) model and mental accounting theory. Integrating these two frameworks, we formally construct an overarching theoretical model: price promotion (Stimulus) → perceived resources (Organism) → consumer food waste behavior (Response), with two theoretically derived boundary conditions that moderate the stimulus-to-organism link. This model provides a robust theoretical lens to explain how price promotions shape consumer food waste behavior and enhances the theoretical visibility of our study.

In the context of this research, we conceptualize price promotion as an external marketing stimulus (S) that alters consumers’ internal cognitive state of perceived resources (O), which then drives their food waste behavioral responses (R). The S-O-R framework allows us to systematically disentangle the causal chain from marketing input to psychological change, and ultimately to waste behavior, addressing the field’s call for more process-focused research on consumer food waste (Block et al., 2016; Schanes et al., 2018).

Second, we draw on mental accounting theory (R. Thaler, 1985) to explain why price promotions amplify consumers’ perceived resources, the core mediating organism in our model. Mental accounting theory argues that consumers do not treat money as perfectly fungible; instead, they categorize financial resources into different mental accounts, evaluate gains and losses relative to reference points, and make consumption decisions based on these subjective mental accounts. A core proposition of this theory is that price promotions create positive transaction utility, where consumers perceive savings by comparing the promotional price to the internal reference price (i.e., the regular price), and this generates a sense of “windfall gain” or psychological income (Arkes et al., 1994; Heilman et al., 2002). Unlike actual income, this windfall gain from promotions is more likely to be coded as a disposable resource in consumers’ mental accounts, which reduces their aversion to wasting resources (R. Thaler, 1985; Zhang et al., 2021). This theory thus provides the micro-foundation for our core mediating mechanism: price promotions enhance perceived resources via changes in consumers’ mental accounting, which subsequently increases food waste behavior.

### 2.2. Core Literature Review

#### 2.2.1. Consumer Food Waste: Drivers of Waste Behavior

As noted in the introduction, consumer food waste is a grand challenge for global sustainable development, with consumer-level disposal accounting for roughly 57% of total food waste across developed and developing economies (Schanes et al., 2018; United Nations, 2023). Academic research has increasingly focused on uncovering the drivers of consumer food waste, which can be broadly categorized into three streams.

The first stream examines individual cognitive and motivational drivers of food waste, predominantly rooted in social psychological theories. For example, studies applying the theory of planned behavior have identified attitudes, subjective norms, and perceived behavioral control as key predictors of household food waste intentions and behaviors (Russell et al., 2017; Stefan et al., 2013). Other work has highlighted the role of consumers’ food safety concerns (Farr-Wharton et al., 2014; Gao, 2025), scarcity mindset (Gao et al., 2024), and aversion to imperfect produce (Mookerjee et al., 2021) in shaping waste decisions. This body of work has advanced our understanding of why consumers waste food, but has largely overlooked how core marketing mix elements (e.g., price promotions) shape waste behavior, despite marketing tools being a primary driver of consumers’ food purchase and consumption decisions (Ilyuk, 2018; Van Doorn, 2016).

The second stream focuses on situational and contextual drivers of consumer food waste. Prior research has documented that social dining contexts (Qian et al., 2021), food product characteristics (Wu et al., 2019), plate size and material (Van Ittersum & Wansink, 2012; Williamson et al., 2016), and macro-level factors such as cultural norms and pandemic-related contextual cues (Gao, 2025) all significantly influence consumers’ food waste decisions. While this research highlights the malleability of food waste behavior to external contexts, it has rarely explored how routine marketing activities (e.g., price promotions) act as a situational trigger for waste. This is a critical gap, especially given the ubiquity of price promotions in food retail and catering settings (Van Lin et al., 2023).

The third stream addresses the measurement of consumer food waste, a critical methodological issue in this field. Existing research employs two primary measurement approaches: actual food waste behavior and food waste intention. Actual behavioral measurement includes objective methods such as weighing leftover food, waste composition analysis, and household waste diaries. These methods have high external validity and ecological realism, but are often costly, make it difficult to control for confounding variables, and have limited ability to isolate causal psychological mechanisms (Aschemann-Witzel et al., 2015; Schanes et al., 2018). In contrast, food waste intention measurement, which is typically via scenario-based scales in experimental settings, captures consumers’ stated likelihood to discard edible food. This type of measurement is widely used in consumer behavior research (Ellison & Lusk, 2018).

Critically, the theory of planned behavior (Ajzen, 1991) and a large body of meta-analytic evidence confirm that behavioral intention is the strongest and most reliable proximal predictor of actual behavior (Sheeran, 2002; Webb & Sheeran, 2006). In food waste research specifically, scenario-based intention measurement allows researchers to strictly control for extraneous factors (e.g., food type, consumption context, household storage conditions) and isolate the causal effect of the target independent variable, which is essential for testing psychological mechanisms (Ellison & Lusk, 2018; Porpino, 2016). Leading marketing journals have consistently published food waste research that combines both intention-based lab experiments and behavioral field studies to triangulate findings, balancing internal validity and external validity (Gao, 2025; Hamerman et al., 2018). In this research, we adopt both measurement approaches: we use scenario-based intention measures to rigorously test the causal chain and boundary conditions in controlled experimental settings, and we use objective weighing of actual food waste in a field setting to confirm the real-world validity of our effects.

#### 2.2.2. Price Promotion: Pre- and Post-Purchase Consumer Behavioral Effects

Price promotions are one of the most pervasive and influential marketing tools used by food retailers, restaurants, and manufacturers worldwide (Ailawadi et al., 2007; Zhang et al., 2021). Extant research on price promotions has predominantly focused on their impacts on consumers’ pre-purchase decision-making, with two core research streams.

The first stream examines the benefits of price promotions for firms and consumers. For firms, price promotions effectively boost short-term sales by accelerating consumer purchase cycles, increasing purchase quantity, and encouraging brand switching (Ailawadi et al., 2007; Bell et al., 1999). For consumers, price promotions deliver both utilitarian benefits (e.g., monetary savings, product upgrading) and hedonic benefits (e.g., positive emotions from “smart shopping”, sense of achievement) (Chandon et al., 2000; Schindler, 1998). This body of work has extensively documented how promotions shape what, when, and how much consumers buy, but has paid limited attention to what happens after consumers make a purchase.

The second stream investigates the potential negative consequences of price promotions. Prior research has found that frequent promotions can erode brand equity, increase long-term consumer price sensitivity, and reduce future sales (Mela et al., 1997; Yoo et al., 2000). Other work has documented that promotions can trigger unplanned and impulse buying, leading to post-purchase guilt (Heilman et al., 2002; Mukhopadhyay & Johar, 2007), and even reduce consumers’ perceived product efficacy (Shiv et al., 2005). However, this line of research has largely overlooked a critical post-purchase outcome: consumer food waste behavior. The few studies that have noted a potential link between promotions and household food waste mostly attribute this effect to promotion-induced over-purchasing (Principato et al., 2021; Van Lin et al., 2023), yet none have empirically unpacked the psychological mechanisms linking price promotions to increased food waste, nor identified boundary conditions to mitigate this unintended negative effect.

Notably, the limited existing work on promotions and food waste has focused almost exclusively on household stockpiling driven by bulk promotions (Van Lin et al., 2023). However, price promotions are also ubiquitous in food service and single-item purchase contexts (e.g., restaurant discounts, retail single-product promotions), where over-purchasing and stockpiling cannot explain post-purchase waste. Our research addresses this gap by examining how price promotions increase food waste even in non-stockpiling contexts, via changes in consumers’ perceived resources, rather than excess purchase quantity.

### 2.3. Research Gap and Research Objectives

Building on the theoretical frameworks and literature review above, we explicitly delineate three critical gaps in the extant literature that our study aims to address, with a tight link to our core research objectives.

First, the literature on consumer food waste has underexplored marketing mix factors as causal antecedents of waste behavior. While prior research has uncovered individual, social, and contextual drivers of food waste, there is a surprising lack of research on how core marketing tools, especially the ubiquitous price promotion, shape consumers’ post-purchase food waste decisions (Block et al., 2016; Van Doorn, 2016). The few studies that have touched on this topic are largely descriptive and correlational, with little attention to causal effects, especially in non-stockpiling contexts. Our research fills this gap by theorizing and empirically testing the causal effect of price promotions on consumer food waste, a critical oversight given that food marketing is a primary driver of consumers’ food-related decisions.

Second, the psychological mechanism underlying the relationship between price promotions and food waste remains undertheorized and untested. Existing research has speculated that over-purchasing and stockpiling may explain why promotions increase household food waste (Brizi & Biraglia, 2021; Van Lin et al., 2023), but this explanation cannot account for waste in non-stockpiling contexts (e.g., restaurant meals, single-item purchases). We address this limitation by identifying perceived resources as the core mediating mechanism. Shaped by mental accounting processes, perceived resources explain why promotions increase food waste even when purchase quantity is held constant. This mechanism also unpacks the micro-psychological process of the social dilemma at the heart of promotional food waste: consumers’ short-term economic gain from promotions (perceived resource abundance) conflicts with the long-term collective interest of reducing environmental harm, leading to increased wasteful behavior.

Third, there is limited research on actionable boundary conditions that can mitigate the food-increasing effect of price promotions. While prior work has identified individual differences that correlate with food waste, there is a lack of research on contextual factors that can be easily manipulated by firms and policymakers to reduce promotion-induced food waste. Our study addresses this gap by examining two theory-driven boundary conditions that directly target the mediating mechanism of perceived resources, offering practical, low-cost interventions to curb the unintended negative consequence of price promotions.

Correspondingly, this research has three core objectives: (1) to establish a causal effect of price promotions on increased consumer food waste behavior, across both intention-based and actual behavioral measures; (2) to unpack the mediating role of perceived resources in explaining this effect; and (3) to identify boundary conditions that attenuate the positive effect of price promotions on food waste, by reducing consumers’ perceived resource abundance from promotions.

### 2.4. Hypotheses Development

#### 2.4.1. Price Promotion and Consumer Food Waste Behavior

We propose that price promotions lead to a significant increase in consumer food waste behavior. Our prediction is rooted in mental accounting theory and the S-O-R framework, as well as prior research on perceived resources and wasteful behavior.

As detailed in the theoretical anchoring section, price promotions generate positive transaction utility for consumers: by comparing the promotional price to the reference price (regular price), consumers perceive a monetary gain from the promotion, which is coded as a windfall gain in their mental accounts (Heilman et al., 2002; R. Thaler, 1985). This windfall gain, even if it does not change consumers’ objective financial resources, significantly enhances their subjective perception of available resources. Perceived resources refer to consumers’ subjective perception of their existing monetary and disposable resources, which is shaped by both objective financial conditions and subjective psychological feelings (Roux et al., 2015; Zhang et al., 2021).

A large body of research has demonstrated that perceived resource abundance is a key driver of wasteful behavior. Consumers are inherently more likely to discard resources when they perceive resources to be plentiful rather than scarce (Roux et al., 2015). Food waste, in particular, has been characterized as a “luxury” behavior that is more prevalent when consumers perceive abundant financial and food resources (Blair & Sobal, 2006; Stancu et al., 2016). For example, cross-country and household-level studies consistently find a positive correlation between income level and consumer food waste: higher-income consumers, who perceive greater resource abundance, generate significantly more food waste than lower-income consumers (Ellison & Lusk, 2018; Parfitt et al., 2010; Xue et al., 2017). Recent experimental research further confirms that a scarcity mindset, which reduces perceived resource abundance, significantly decreases consumers’ food waste behavior, even when the actual amount of food is held constant (Gao et al., 2024).

Building on this literature, we argue that price promotions enhance consumers’ perceived resources, which in turn reduces the perceived psychological and economic cost of discarding edible food, leading to higher levels of food waste. Importantly, this effect is independent of over-purchasing or stockpiling: even when consumers purchase the same quantity of food, the subjective feeling of resource abundance from the promotion makes them more willing to discard edible food that does not meet their expectations (e.g., food with suboptimal taste, leftovers). Thus, we propose our first two hypotheses:

**H1.**  *Price promotions significantly increase consumer food waste behavior.*

**H2.**  *Perceived resources mediate the positive effect of price promotions on consumer food waste behavior.*

#### 2.4.2. Boundary Conditions of the Price Promotion-Food Waste Effect

Our theorizing suggests that the positive effect of price promotions on food waste is driven by enhanced perceived resources, which stems from consumers’ focus on the monetary savings generated by the promotion. If this mechanism holds, the effect of price promotions on food waste should be attenuated when factors that reduce consumers’ perceived resource abundance from promotions are activated. We examine two theory-driven boundary conditions that directly target the core mediating mechanism: consumers’ attentional focus (on money spent vs. money saved) and budget consciousness.

The subjective perception of resource gain from price promotions depends critically on consumers’ attentional focus: the mental accounting benefit of promotions is most salient when consumers focus on the money they saved relative to the reference price (Lichtenstein et al., 1990; R. Thaler, 1985). In contrast, when consumers’ attention is directed to the money they have spent (rather than saved), the windfall gain perception from the promotion is diminished, and they are more likely to recognize that the purchase reduces their available monetary resources, rather than increasing their disposable resources (Heilman et al., 2002; Zhang et al., 2021).

Prior research shows that shifting consumers’ focus from gains to losses can significantly alter their mental accounting and subsequent consumption decisions (R. H. Thaler, 1999). For example, consumers are less likely to spend windfall gains when they are prompted to focus on their overall budget constraints, rather than the gain itself (Arkes et al., 1994). In the context of price promotions, when consumers are directed to focus on their expenditure (the money spent on the purchase), the subjective feeling of resource abundance from the promotion will be weakened, which in turn reduces their willingness to waste food. In contrast, when consumers focus on the money saved from the promotion, the positive effect of promotions on perceived resources and subsequent food waste will remain robust. Thus, we hypothesize:

**H3.**  *The positive effect of price promotions on consumer food waste behavior is attenuated when consumers focus on the amount of money spent (vs. money saved) from the transaction.*

We further propose that consumers’ psychological budget constraints act as a second boundary condition. Mental accounting theory posits that consumers set mental budgets for different spending categories, and use these budgets as reference points to evaluate their spending decisions (Heath & Soll, 1996; R. Thaler, 1985; Stilley et al., 2010). The perception of resource abundance from price promotions is contingent on consumers’ perceived standing relative to their mental budget: when consumers perceive that their spending is within their pre-set budget, the savings from promotions are more likely to be coded as a disposable windfall gain, enhancing perceived resources. However, when consumers perceive that their spending has exceeded their mental budget, the savings from the promotion will be offset by the loss of overspending, and the perception of resource abundance will be eliminated (Stilley et al., 2010; Zhang et al., 2021).

Extant research confirms that exceeding a mental budget triggers a sense of financial constraint, which reduces consumers’ willingness to engage in wasteful or discretionary spending (Cheema & Soman, 2006). In the context of food waste, consumers who perceive they have overspent their budget will have lower levels of perceived resources, regardless of any savings from promotions, which will reduce their tendency to waste edible food. In contrast, for consumers who spend within their budget, price promotions will significantly enhance perceived resources, leading to increased food waste. Therefore, we hypothesize:

**H4.**  *The positive effect of price promotions on consumer food waste behavior is attenuated when consumers perceive their spending as exceeding (vs. being within) their psychological budget.*

**Figure 1:**
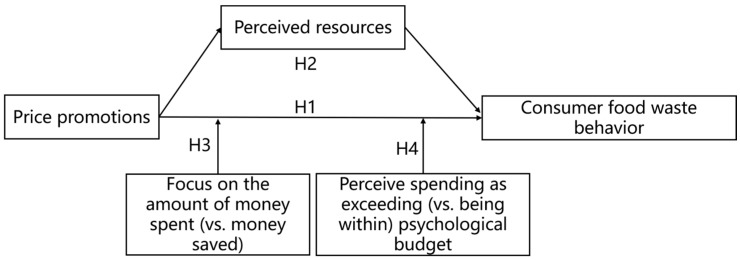
The conceptual research model.

Figure 1 presents the conceptual research model.

## 3. Method

### 3.1. Study 1

In Study 1, the objective is to establish support regarding the influence of discounts on customers’ food waste behavior through the manipulation of both the existence and the degree of these promotions. Moreover, an in-depth investigation is carried out to explore how perceived resources serve as a mediating factor in the influence of price promotions on customers’ food waste behavior.

#### 3.1.1. Design and Procedure

We conducted an a priori sample size calculation using G*Power 3.1 for a one-way ANOVA with three independent groups. Setting α = 0.05, statistical power (1 − β) = 0.90, and a medium effect size (f = 0.30), the minimum required sample size was 158. To account for potential inattentive responses, we recruited 195 participants, which exceeded the required sample size and ensured sufficient statistical power for hypothesis testing. We recruited 195 participants from the Chinese panel of the survey platform Credamo to complete the study, and all participants received monetary compensation upon completion. The participants included 129 women (66.15%). The average age of the samples was 28.8 years. This gender distribution reflects the platform’s user structure (predominantly young female users), rather than intentional sampling bias. Participants were randomly assigned to one of three between-subjects conditions via computer-generated randomization: no-promotion group, 20%-off promotion group, or 50%-off promotion group. Random assignment ensured group equivalence at the baseline.

Each participant was instructed to imagine themselves going to a shopping center. Under the condition of no promotion, participants were only informed that they had spent 1000 yuan on the items. Under the condition of a 20% discount promotion, participants were informed that the mall was undergoing a 20% discount promotion, and they spent 1000 yuan to purchase goods that were originally worth 1200 yuan. Under the condition of a 50% discount promotion, participants were informed that the mall was conducting a 50% discount promotion, and they spent 1000 yuan to purchase products that were originally worth 2000 yuan. Subsequently, all participants read an identical food disposal scenario to trigger food waste intention, a common paradigm in consumer food waste research (Ilyuk, 2018). Participants were required to imagine a situation where they returned home from the mall and opened a box of cookies purchased from the mall. However, the taste of cookies was not what they expected, the cookie tasted bitter, thus they were a little disappointed with the cookies they bought. Participants were then asked to indicate their waste-related intentions using three items (Williamson et al., 2016; Russell et al., 2017): “How likely are you to throw these cookies away,” “How likely are you to continue eating the cookies(reverse-coded),” and “How likely are you to keep the cookies(reverse-coded)” (1 = very unlikely, 7 = very likely). The items were averaged to form a food waste intention index, with higher scores indicating greater willingness to waste food. The scale demonstrated excellent internal consistency (Cronbach’s α = 0.87). Next, participants answered three items on perceived resources: “After shopping, I feel I have more resources,” “After shopping, I feel I have sufficient resources,” and “After shopping, I feel I have saved a lot of money” (1 = strongly disagree, 7 = strongly agree). The items were averaged to form a perceived resources index, with higher scores indicating a stronger sense of available monetary resources. The scale had high internal consistency (Cronbach’s α = 0.91). In the end, participants completed a demographic questionnaire (gender, age, household income, frequency of food purchase) and were debriefed and compensated.

#### 3.1.2. Results and Discussion

**Figure 2:**
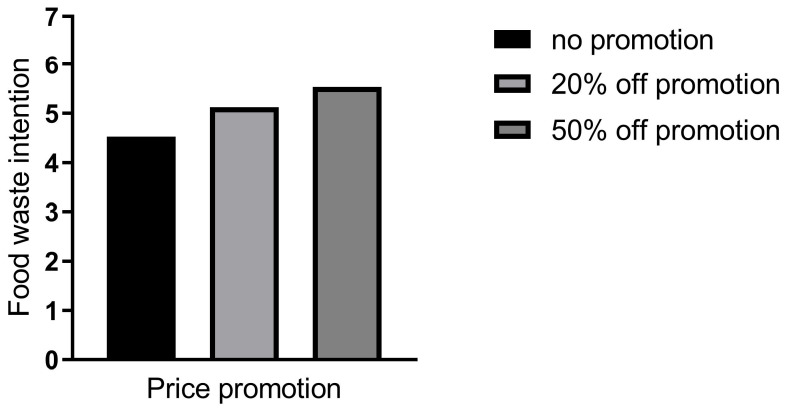
The influence of price promotion on consumer food waste intention (Study 1).

In Study 1, the independent variable was price promotion, and the dependent variable was consumers’ food waste intention. An ANOVA model was utilized to test the samples. The statistical analysis showed that the main effect was significant (F (2, 192) = 10.42, *p* < 0.01, ƞ^2^ = 0.098). As depicted in Figure 2, participants in the 50%-off condition were more inclined to engage in food waste compared to those in the no-price-promotion condition (M_50%-off_ = 5.53 vs. M_no-promotion_ = 4.52, SD_50%-off_ = 1.12 vs. SD_no-promotion_ = 1.34; *t* (192) = 4.54, *p* < 0.01; *d*_cohen_ = 0.80). Additionally, participants in the 20%-off condition demonstrated a significantly higher food waste intention than those in the no-price-promotion condition (M_20%-off_ = 5.12 vs. M_no-promotion_ = 4.52, SD_20%-off_ = 1.31 vs. SD_no-promotion_ = 1.34; *t* (192) = 2.71, *p* = 0.02; *d*_cohen_ = 0.48). However, there was no significant difference in consumers’ food waste intention between the 20%-off condition and the 50%-off condition (*t* (192) = 1.83, *p* = 0.16; *d*_cohen_ = 0.32). Theoretically, this non-significant result suggests a threshold effect: any level of price promotion (vs. no promotion) triggers a significant increase in food waste intention, but increasing the discount depth beyond a certain threshold (20% in this study) does not produce an additional incremental effect on waste intention. Critically, this result does not challenge H1, as both discount conditions exhibited significantly higher food waste intention than the no-promotion control group.

**Figure 3:**
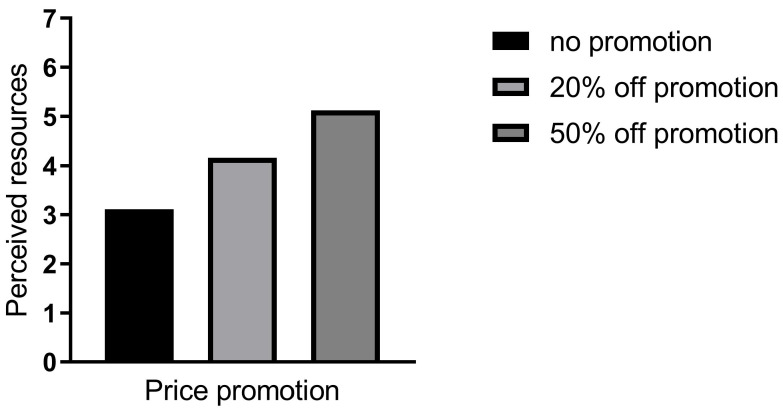
The influence of price promotion on perceived resources (Study 1).

To examine the mediation effect, the price promotion was the independent variable, and the perceived resources was set as the dependent variable. An ANOVA model was then employed to conduct sample analysis. The statistical analysis showed that the main effect was significant (F (2, 192) = 74.57, *p* < 0.01, ƞ^2^ = 0.44). As illustrated in Figure 3, participants in the 50%-off condition showed significantly higher levels of perceived resources compared to those in the no-promotion condition (M_50%-off_ = 5.86 vs. M_no-promotion_ = 3.11, SD_50%-off_ = 0.75 vs. SD_no-promotion_ = 1.29; *t* (192) = 12.101, *p* < 0.01; *d*_cohen_ = 2.12). Similarly, participants in the 20%-off condition demonstrated a significantly greater sense of perceived resources than those in the no-price-promotion condition (M_20%-off_ = 4.16 vs. M_no-promotion_ = 3.11, SD_20%-off_ = 1.68 vs. SD_no-promotion_ = 1.29; *t* (192) = 4.63, *p* < 0.01; *d*_cohen_ = 0.81). Furthermore, participants in the 50%-off condition also indicated higher levels of perceived resources than those in the 20%-off condition (M_20%-off_ = 4.16 vs. M_50%-off_ = 5.12, SD_20%-off_ = 1.68 vs. SD_50%-off_ = 1.31 *t* (192) = 7.47, *p* < 0.01; *d*_cohen_ = 1.31). This result confirms that price promotion significantly increases consumers’ perceived resources, and that discount depth has a positive linear effect on perceived resources (deeper discounts = higher perceived resources).

Subsequently, the SPSS 24.0 PROCESS macro was utilized to analyze mediation effect. A sample size of 5000 was selected, and Model 4 was employed. The price promotion was treated as a multi-categorical variable, the perceived resources as the mediating variable, and the consumers’ food waste intention as the dependent variable. The results demonstrated that the indirect effect of the 20%-off promotion on consumers’ food waste intention was 0.33 (CI_95%_ = [0.131, 0.602], not containing zero). This indicates that the indirect effect was statistically significant. In contrast, the direct effect of the 20%-off promotion on consumers’ food waste intention was 0.27 (CI_95%_ = [−0.170, 0.703], including zero), suggesting that the direct effect was not significant. Moreover, the results indicated that the indirect effect of the 50%-off promotion on consumers’ food waste intention was 0.87 (CI_95%_ = [0.465, 1.313], not including zero), signifying that the indirect effect was significant. The direct effect of the 50%-off promotion on consumers’ food waste intention was 0.13 (CI_95%_ = [−0.416, 0.683], including zero), indicating that the direct effect was not significant.

In summary, Study 1 provides robust evidence for H1 and H2: price promotion significantly increases consumers’ food waste intention, and this effect is fully mediated by perceived resources. The threshold effect of discount depth on food waste intention further suggests that the psychological perception of “saving money” (rather than the magnitude of savings) is the key driver of the focal effect.

### 3.2. Study 2

Study 2 aimed to replicate the main effect of price promotion on food waste behavior (H1) in a real-world field experiment using objective behavioral measures (rather than self-reported intention). This study addressed the limitation of scenario-based experiments and enhanced the external validity of our findings.

#### 3.2.1. Design and Procedure

We conducted this field experiment in April 2023 at a university cafeteria in Changchun, China. This cafeteria serves more than 2000 individual diners daily, with fully standardized food portion sizes and pricing. We employed a one-factor between-subjects design with two conditions (price promotion vs. no promotion) and randomly assigned eligible diners to one of the two groups.

We set explicit inclusion and exclusion criteria to ensure the internal validity of our food waste measurement. Specifically, we only included diners who placed individual food orders and dined on-site, to eliminate confounding effects from group orders and takeout orders on our core dependent variables. Diners who shared food with others, or who did not finish their meal due to time constraints, were excluded from the final sample. Data collection spanned a 12 h window from 8:00 a.m. to 8:00 p.m. on the experiment day, yielding a final valid sample of 166 diners, with 83 randomly assigned to the price promotion condition and 83 to the no-promotion control condition. We collected 166 valid samples (83 per group), achieving a post hoc power of 0.87 for the observed effect size (d = 0.39), which meets the conventional 0.80 threshold for statistical power.

To eliminate observer bias and ensure the objectivity of the experiment, we implemented a rigorous double-blind design with two entirely independent teams of research assistants, who had no communication with each other throughout the entire study. The manipulation team was stationed at the cashier desk, responsible for executing the experimental manipulation and random assignment. For diners assigned to the price promotion condition, the cashier informed them that they would receive an instant 10 RMB discount for their current order upon payment. Diners in the no-promotion condition received no discount or coupon during checkout. Random assignment was implemented via a pre-generated random number sequence, and the cashier alternated condition assignments strictly following this sequence to avoid selection bias. Critically, the entire manipulation was seamlessly integrated into the routine checkout process, and diners remained unaware of their participation in an experiment.

The measurement team was positioned at the tray return station, and remained fully blind to participants’ condition assignment throughout the experiment, with no access to information about whether a diner had received a discount. All members of the measurement team completed a 4 h standardized training session prior to the experiment, covering standardized weighing protocols and data recording specifications. All food waste measurements were conducted using calibrated high-precision electronic scales with an accuracy of ±1 g.

We followed a standardized three-step measurement procedure to capture objective food waste outcomes. First, we conducted the pre-consumption measurement: before diners took their meals, we weighed the gross weight of the food container plus the food (W1), and recorded the pre-measured net weight of the corresponding standardized empty container (W0). Second, we completed the post-consumption measurement immediately after diners finished their meals and returned their trays, weighing the gross weight of the container with leftover food (W2). Based on these three data points, we constructed two objective indicators of food waste behavior to rule out the influence of differences in initial portion size: the absolute amount of food waste (in grams), calculated as W2 minus W0, and the food waste rate (in percentage), calculated as [(W2 − W0)/(W1 − W0)] × 100%.

To ensure data quality, all weight measurements were double-recorded by two independent research assistants. Any discrepancy of 5 g or more between the two recordings was resolved by immediate re-weighing. Without direct interaction with diners, research assistants also recorded diners’ gender, estimated age, and number of dining companions, which were included as control variables in supplementary robustness analyses.

#### 3.2.2. Results and Discussion

Firstly, the effect of discount on the amount of food waste was examined. Price promotion was designated as the independent variable (0 representing no price promotion and 1 representing price promotion), and the amount of food waste was set as the dependent variable. Based on a *t*-test, consumers in the promotion condition generated a significantly larger amount of food waste than those in the no-promotion condition (M_promotion_ = 145.26 vs. M_no-promotion_ = 115.03, SD_promotion_ = 75.14 vs. SD_no-promotion_ = 80.87; *t* (164) = 2.495, *p* = 0.014; *d*_cohen_ = 0.387). Furthermore, the effect of price promotion on the food waste rate was investigated. A *t*-test further demonstrated that consumers in the promotion condition had a significantly higher food waste rate than those in the no-promotion condition (M_promotion_ = 30.6% vs. M_no-promotion_ = 24.9%, SD_promotion_ = 0.16 vs. SD_no-promotion_ = 0.18; *t* (164) = 2.182, *p* = 0.031; *d*_cohen_ = 0.339).

Study 2 provides causal real-world evidence for H1, replicating the findings of Study 1 with objective behavioral measures. The double-blind design and standardized measurement procedures eliminate observer bias and measurement error, validating that price promotion not only increases consumers’ food waste intention but also their actual food waste behavior. The significant effect on food waste rate further indicates that the promotion effect is not driven by larger food purchases, but by a higher proportion of purchased food being wasted—consistent with our theoretical mechanism of perceived resources.

### 3.3. Study 3

Study 3 aimed to test the moderating role of spending focus (money-saving focus vs. money-spending focus) in the relationship between price promotion and food waste intention (H3). This study further validates our theoretical mechanism by examining whether shifting consumers’ attention from savings to spending attenuates the effect of price promotion on food waste intention (by reducing perceived resources).

#### 3.3.1. Design and Procedure

A total of 284 undergraduate students from a comprehensive university in China completed this online experiment in exchange for 20 RMB in monetary compensation. We conducted an a priori sample size calculation using G*Power 3.1 for a 2 × 2 between-subjects ANOVA, focusing on the interaction effect (the key test for H3). Setting α = 0.05, power (1 − β) = 0.90, and a medium effect size for the interaction (f = 0.25), the minimum required sample size was 210. We recruited 284 participants to account for inattentive responses, ensuring sufficient statistical power for the interaction effect test. Eligible participants were required to have prior experience purchasing and disposing of fast food, to pass an embedded attention check, and to have not participated in any of our other related studies. The final valid sample comprised 188 women (66.19%) and 96 men (33.81%), with a mean age of 20.16 years (SD = 0.82, range 19–22 years). This gender distribution reflects the demographic structure of the university’s undergraduate student population, which is predominantly composed of young female students, rather than intentional sampling bias. Participants were randomly assigned to one of four experimental conditions using a computer-generated randomization procedure, corresponding to a 2 (price promotion: present vs. absent) × 2 (spending focus: money-saving vs. money-spending) between-subjects factorial design.

Participants first read a scenario describing the purchase of a voucher for well-known fast-food restaurants located near the campus, either McDonald’s or Burger King. We manipulated price promotion between subjects: in the no-promotion condition, participants were informed that they spent 10 RMB to purchase a 10 RMB voucher for the restaurant, with no associated savings. In the price promotion condition, participants learned that they could spend 10 RMB to obtain a 20 RMB voucher for the same restaurant, representing a 50% discount aligned with the deep discount condition used in Study 1.

Immediately following the price promotion manipulation, we manipulated spending focus using a directed recall task. In the money-saving focus condition, participants were prompted to recall and write down the exact amount of money they saved by purchasing the voucher. In the money-spending focus condition, participants were instead prompted to recall and write down the exact amount of money they spent to purchase the voucher. To verify that participants experienced the intended psychological states from the spending focus manipulation, we included two manipulation check items. All items were measured on a 7-point Likert scale anchored at 1 = strongly disagree and 7 = strongly agree, with statements reading “When thinking about the voucher purchase, I focused more on the money I saved” and “When thinking about the voucher purchase, I focused more on the money I spent.” The two items were averaged to form a spending focus index, which demonstrated excellent internal consistency (Cronbach’s α = 0.85), with higher scores indicating a stronger money-saving focus and lower scores indicating a stronger money-spending focus.

After completing the manipulation check, all participants read an identical food disposal scenario consistent with the one used in Study 1, which described ordering a pizza that tasted bitter and failed to meet their expectations upon delivery. Our core dependent variable, food waste intention, was measured using the same validated 3-item scale employed in Study 1 (Williamson et al., 2016; Russell et al., 2017). All items were rated on a 7-point Likert scale, with reverse coding applied to items assessing intentions to continue eating or keep the food, and the scale exhibited excellent internal consistency (Cronbach’s α = 0.89). At the end of the experiment, participants completed a brief demographic questionnaire before being fully debriefed about the study’s purpose.

#### 3.3.2. Results and Discussion

We first evaluated the effectiveness of our spending focus manipulation. Results from an independent-samples *t*-test confirmed that our manipulation was successful: participants in the money-saving focus condition reported significantly higher scores on the spending focus index (M = 5.82, SD = 0.97) than those in the money-spending focus condition (M = 2.31, SD = 1.05), *t* (282) = 28.76, *p* < 0.001, *d*_cohen_ = 3.42. To test our hypothesis that spending focus moderates the effect of price promotion on food waste intention (H3), we conducted a 2 (price promotion: present vs. absent) × 2 (spending focus: money-saving vs. money-spending) between-subjects analysis of variance (ANOVA), with food waste intention as the dependent variable. The ANOVA revealed no significant main effect of price promotion, F(1, 280) = 2.37, *p* = 0.125, η^2^ = 0.008, nor a significant main effect of spending focus, F(1, 280) = 3.39, *p* = 0.067, η^2^ = 0.012. Critically, and aligned with our theoretical predictions, the two-way interaction between price promotion and spending focus was statistically significant, F(1, 280) = 8.40, *p* < 0.01, η^2^ = 0.03, providing direct support for H3.

We unpacked this significant interaction using pre-specified simple effect analyses guided by our theoretical framework. When participants were directed to focus on the money saved from the transaction, we replicated our core main effect: participants in the price promotion condition reported significantly greater food waste intention than those in the no-promotion condition (M_promotion_ = 5.29 vs. M_no-promotion_ = 4.61, SD_promotion_ = 1.17 vs. SD_no-promotion_ = 1.36; *t* (280) = 3.14, *p* = 0.01; *d*_cohen_ = 0.53). In stark contrast, when participants were directed to focus on the money spent in the transaction, the effect of price promotion on food waste intention was completely attenuated. There was no significant difference in food waste intention between the price promotion and no-promotion conditions in this case (M_promotion_ = 4.56, vs. M_no-promotion_ = 4.77, SD_promotion_ = 1.41 vs. SD_no-promotion_ = 1.29; *t* (280) = 0.96, *p* = 0.77; *d*_cohen_ = 0.16).

Taken together, the findings from Study 3 provide robust empirical support for H3, demonstrating that consumers’ attentional focus on spending versus savings serves as a critical boundary condition for the effect of price promotions on food waste intention. Specifically, shifting consumers’ attention away from the savings gained through a promotion and toward the money spent eliminates the promotion-induced increase in food waste intention. This pattern of results aligns perfectly with our proposed underlying mechanism: the effect of price promotions on food waste operates through expanded perceptions of available resources, a psychological state that is erased when consumers focus on their expenditures rather than their savings. These findings further reinforce that perceived resources are the key psychological driver of the link between price promotions and increased consumer food waste.

### 3.4. Study 4

Study 4 aimed to test the moderating role of budget status (spending within budget vs. spending over budget) in the relationship between price promotion and food waste intention (H4). This study identifies another critical boundary condition for the focal effect and provides convergent evidence for our theoretical framework: activating consumers’ budget consciousness attenuates the effect of price promotion on food waste intention.

#### 3.4.1. Design and Procedure

A total of 324 Chinese undergraduate students were recruited from a professional online participant pool, with each participant receiving 10 RMB in monetary compensation upon completion of the study. We conducted an a priori sample size calculation using G*Power 3.1 for a 2 × 2 between-subjects ANOVA, focusing on the interaction effect (the key test for H4). Setting α = 0.05, power (1 − β) = 0.90, and a medium effect size for the interaction (f = 0.25), the minimum required sample size was 210. We recruited 324 participants to account for inattentive responses, ensuring sufficient statistical power for hypothesis testing. Eligible participants were required to be the primary decision-maker for their personal consumption, have prior experience with online shopping and food disposal behaviors, pass an embedded attention check, and have not participated in any of our earlier related studies. The final valid sample consisted of 217 women (66.98%) and 107 men (33.02%), with a mean age of 19.75 years (SD = 1.02, range 18–23 years). This gender distribution aligns with the core user structure of the recruitment platform, which is predominantly composed of young female students, rather than stemming from intentional sampling bias. Participants were randomly assigned to one of four experimental conditions through a computer-generated randomization procedure, corresponding to a 2 (price promotion: absent vs. present) × 2 (budget status: within budget vs. over budget) between-subjects factorial design.

All participants first read a hypothetical online shopping scenario set in their favorite retail store, where we manipulated the presence of price promotion between subjects. In the no-promotion condition, participants were informed that they had spent 500 RMB on items from the store, with no ongoing promotional activities. In the price promotion condition, participants learned that they had spent 500 RMB to purchase items with an original price of 1000 RMB, representing a 50% discount consistent with the deep discount manipulation used in Studies 1 and 3.

Following the price promotion manipulation, we manipulated participants’ perceived budget status using a well-established paradigm from psychological budgeting research. In the within-budget condition, participants were informed that the 500 RMB expenditure fell within their pre-set monthly shopping budget. In the over-budget condition, participants were told that the 500 RMB expenditure had exceeded their pre-set monthly shopping budget.

To verify that participants perceived the intended budget status from our manipulation, we included two manipulation check items. All items were rated on a 7-point Likert scale anchored at 1 = strongly disagree and 7 = strongly agree, with statements reading “I feel that this 500 RMB expenditure is within my monthly shopping budget” and “I feel that this 500 RMB expenditure has exceeded my monthly shopping budget (reverse-coded).” The two items were averaged to form a budget status index, which demonstrated excellent internal consistency (Cronbach’s α = 0.92), with higher scores indicating a stronger perception of being within budget and lower scores reflecting a stronger perception of overspending.

After completing the manipulations checks, all participants read an identical food disposal scenario consistent with the paradigms used in Studies 1 and 3, which described purchasing a box of cookies that tasted bitter and failed to meet their expectations upon delivery. Our core dependent variable, food waste intention, was measured using the same validated 3-item scale employed in our prior studies (Williamson et al., 2016; Russell et al., 2017). All items were rated on a 7-point Likert scale, with reverse coding applied to items assessing intentions to continue eating or keep the cookies, and the scale exhibited excellent internal consistency (Cronbach’s α = 0.88). At the conclusion of the experiment, participants completed a brief demographic questionnaire before being fully debriefed about the study’s purpose.

#### 3.4.2. Results and Discussion

We first assessed the effectiveness of our budget status manipulation. Results from an independent-samples *t*-test confirmed that the manipulation was highly effective: participants in the within-budget condition reported significantly higher scores on the budget status index (M = 6.01, SD = 0.89) than those in the over-budget condition (M = 2.15, SD = 1.02), *t* (322) = 34.57, *p* < 0.001, *d*_cohen_ = 4.01.

To test our hypothesis that perceived budget status moderates the effect of price promotion on food waste intention (H4), we conducted a 2 (price promotion: absent vs. present) × 2 (budget status: within budget vs. over budget) between-subjects analysis of variance (ANOVA), with food waste intention as the dependent variable. The ANOVA revealed no statistically significant main effect of price promotion, F(1, 320) = 1.91, *p* = 0.17, η^2^ = 0.006, nor a significant main effect of budget status, F(1, 320) = 2.06, *p* = 0.15, η^2^ = 0.006. Critically, and aligned with our theoretical predictions, the two-way interaction between price promotion and budget status was statistically significant, F(1, 320) = 5.34, *p* = 0.02, η^2^ = 0.016, providing direct support for H4.

We unpacked this significant interaction using theory-driven simple effect analyses. When participants were told their expenditure fell within their pre-set monthly budget, we replicated our core main effect: participants in the price promotion condition reported significantly greater food waste intention than those in the no-promotion condition (M_promotion_ = 5.12 vs. M_no-promotion_ = 4.57, SD_promotion_ = 1.33 vs. SD_no-promotion_ = 1.36; *t* (320) = 2.61, *p* = 0.046; *d*_cohen_ = 0.41). In stark contrast, when participants were informed that their spending had exceeded their monthly budget, the effect of price promotion on food waste intention was fully attenuated. There was no significant difference in food waste intention between the price promotion and no-promotion conditions in this case (M_promotion_ = 4.56 vs. M_no-promotion_ = 4.70, SD_promotion_ = 1.38 vs. SD_no-promotion_ = 1.34; *t* (320) = 0.658, *p* = 0.91; *d*_cohen_ = 0.103).

Taken together, the results of Study 4 provide robust empirical support for H4, demonstrating that consumers’ perceived budget status serves as a critical boundary condition for the effect of price promotions on food waste intention. Specifically, when consumers perceive that their spending has exceeded their psychological budget, the promotion-induced expansion of perceived resources is eliminated, which in turn attenuates the positive effect of price promotions on food waste intention. This finding further validates our overarching theoretical framework, identifying budget consciousness as a second key boundary condition alongside the attentional focus documented in Study 3. In tandem, these moderation results provide convergent evidence for our proposed mediating mechanism, reinforcing that perceived resources are the core psychological driver linking price promotions to increased consumer food waste.

## 4. General Discussion

Global food waste at the consumer level represents a defining social dilemma of sustainable consumption, wherein individual pursuit of short-term economic benefits inflicts irreversible long-term harm on collective environmental and social welfare. Across four complementary studies, including three controlled scenario-based experiments and one real-world field experiment, we provide robust, convergent causal evidence that price promotions, one of the most ubiquitous marketing tools in the food industry, significantly increase consumers’ food waste behavior. In line with our theoretical framework anchored in the Stimulus–Organism–Response (S-O-R) model and mental accounting theory, we find that price promotions enhance consumers’ perceived resources, which in turn drive greater willingness to discard edible food and higher levels of actual food waste. We further delineate two actionable boundary conditions that mitigate this unintended pro-waste effect: the effect of price promotions on food waste is eliminated when consumers focus on the money they have spent (rather than the money saved) from the transaction, and when consumers perceive their spending as exceeding their pre-set psychological budget. Taken together, our findings unpack the black box of the psychological mechanism linking price promotions to consumer food waste, address longstanding calls for marketing-focused research on the drivers of food waste (Block et al., 2016; Van Doorn, 2016), and offer theoretically grounded, actionable insights for advancing sustainable consumption.

### 4.1. Theoretical Contributions

This research makes three core theoretical contributions to the literature on price promotions, consumer food waste, and sustainable consumption decision-making.

First, our work extends the long-standing price promotion literature by shifting scholarly attention from pre-purchase decision-making to understudied post-purchase behavioral consequences, particularly those related to sustainability. Decades of marketing research have extensively documented the effects of price promotions on firms’ sales performance and consumers’ pre-purchase decisions, including purchase acceleration, impulse buying, and brand switching (Ailawadi et al., 2007; Bell et al., 1999). While a small body of work has identified negative post-purchase outcomes of promotions, such as reduced perceived product efficacy (Shiv et al., 2005), post-purchase guilt (Mukhopadhyay & Johar, 2007), and increased impatience in unrelated domains (Shaddy & Lee, 2020), our research is the first to systematically establish a causal link between price promotions and consumers’ post-purchase food waste behavior. Critically, we advance beyond the prevailing correlational speculation that promotions increase food waste solely via over-purchasing and stockpiling (Brizi & Biraglia, 2021; Van Lin et al., 2023). By holding purchase quantity constant across experimental conditions, we demonstrate that price promotions increase food waste even in the absence of stockpiling, via changes in consumers’ subjective psychological state. This finding reveals a previously undocumented hidden cost of price promotions, broadening the nomological network of promotional effects to include environmentally consequential post-purchase behaviors, and answering repeated calls for research into the sustainability implications of core marketing mix strategies (Mookerjee et al., 2021; Van Doorn, 2016).

Second, our study advances the consumer food waste literature by identifying and empirically validating a novel psychological mechanism that explains the micro-foundations of food waste decisions. Extant research on food waste drivers has predominantly focused on either stable individual differences (e.g., attitudes, norms) or objective contextual factors (e.g., social dining, food characteristics), with limited attention to the malleable cognitive processes triggered by routine marketing activities (Gao et al., 2024; Schanes et al., 2018; Stefan et al., 2013). We address this gap by theorizing and documenting the mediating role of perceived resources, a subjective cognitive state shaped by mental accounting processes. Specifically, we show that price promotions generate a sense of windfall gain via transaction utility (R. Thaler, 1985), which enhances consumers’ perceived resource abundance even when their objective financial resources remain unchanged. This subjective perception of resource plenty, in turn, lowers the psychological cost of discarding edible food, driving greater waste. This finding contributes to the literature by disentangling the effect of subjective perceived resources from objective resource availability, which has been the primary focus of prior food waste research (Ellison & Lusk, 2018; Xue et al., 2017). In doing so, we unpack the micro-psychological process underlying the classic social dilemma in sustainable consumption: we explain how a marketing tool that delivers immediate individual economic benefits can inadvertently exacerbate collective environmental harm, by reshaping consumers’ perception of their own resources.

Third, our research strengthens the causal understanding of consumer food waste behavior, addressing a key methodological limitation in the extant literature. Prior research on consumer food waste has largely relied on correlational designs, cross-sectional surveys, and descriptive analyses, which are limited in their ability to establish causal effects and isolate underlying psychological mechanisms (Porpino, 2016; Van Doorn, 2016). In contrast, our research combines controlled experiments with rigorous causal identification and a field experiment with high ecological validity. The scenario-based experiments allow us to strictly control for confounding factors (e.g., purchase quantity, food type, storage conditions) and isolate the causal effect of price promotions on food waste via the proposed psychological mechanism. The field experiment in a real-world canteen setting complements the experimental findings by documenting the effect on actual food waste behavior, rather than just stated intentions. By triangulating across multiple methods, samples, and consumption contexts, we provide robust causal evidence for the effect of marketing activities on food waste, setting a foundation for future experimental research on the psychological drivers of sustainable (and unsustainable) consumption.

### 4.2. Practical Implications

The global challenge of reducing consumer food waste requires coordinated action across multiple stakeholders, including policymakers, food retailers, food service operators, and non-governmental organizations. Our findings offer nuanced, actionable, and incentive-compatible implications for each of these stakeholders, addressing the tension between commercial objectives and sustainability goals, and providing targeted strategies to mitigate promotion-induced food waste.

First, for national and local policymakers and sustainability regulators, our findings provide evidence-based guidance for designing targeted interventions to curb consumer food waste. Our core finding that price promotions are a significant trigger of food waste identifies high-priority windows for policy intervention: periods of concentrated promotional activity, such as China’s Singles’ Day shopping festival, the U.S. Black Friday and Cyber Monday sales, and year-end holiday promotions. During these periods, policymakers can prioritize resources for public awareness campaigns that reframe consumer attention from promotional savings to overall spending and budget constraints. For example, public service announcements can educate consumers about the hidden environmental cost of promotional purchases, and provide simple nudges to help consumers track their total spending rather than just the money saved from individual discounts. Additionally, regulators can work with industry associations to develop voluntary or mandatory guidelines for food retailers and caterers, such as mandating the prominent display of total spending information alongside discount savings in promotional materials, to activate consumers’ budget consciousness at the point of purchase. These interventions are low-cost, scalable, and directly target the psychological mechanism we identify, without restricting businesses’ ability to use promotional tools to drive economic activity.

Second, for food retailers and fast-moving consumer goods (FMCG) brands, our findings offer a path to mitigate the unintended environmental costs of price promotions while aligning with core business objectives, including brand equity building, customer loyalty, and environmental, social, and governance (ESG) performance. Contrary to the apparent tension between promoting sales and reducing waste, our insights enable retailers to design promotions that balance commercial and sustainability goals. For example, instead of broad-based price discounts that amplify perceived resource abundance and subsequent waste, retailers can design promotions that are tied to waste-reduction behaviors, such as discounts for consumers who demonstrate consistent low food waste via loyalty program tracking, or bundle promotions that pair discounted staple foods with food storage and preservation products. Furthermore, retailers can integrate subtle, non-intrusive nudges into the shopping journey that activate budget consciousness without discouraging purchases: for example, displaying the total amount spent in the shopping cart in a prominent position on e-commerce platforms and in-store self-checkout systems, rather than only highlighting the total amount saved from discounts. These nudges directly mitigate the pro-waste effect of promotions by shifting consumers’ attentional focus from savings to spending, while also supporting consumers’ own financial planning goals, which can enhance long-term customer satisfaction and loyalty. In addition, documenting and communicating efforts to reduce promotion-induced food waste can strengthen brands’ ESG credentials, which is increasingly valued by investors, regulators, and sustainability-focused consumers.

Third, for food service operators (including workplace and university canteens, restaurants, and catering services), our findings provide actionable strategies to reduce plate waste in promotional settings. Our field experiment confirms that even small, immediate discounts in a canteen setting significantly increase consumers’ actual food waste. For canteens and casual dining operators, this means that promotional activities (e.g., discount coupons, meal deals) should be paired with targeted waste-reduction interventions. For example, when distributing discount coupons, operators can include a gentle reminder of consumers’ food budget, or tie discounts to the “clean plate” initiative, such as offering additional loyalty points for consumers who finish their meals. For restaurants, promotional set menus can be designed with flexible portion options, allowing consumers to adjust serving sizes while still accessing the promotional discount, reducing waste from over-serving. These strategies not only reduce food waste and associated operational costs (e.g., food procurement and waste disposal costs), but also align with consumers’ growing preference for sustainable dining options.

### 4.3. Limitations and Future Research Directions

While our four studies provide robust causal evidence for the effect of price promotions on consumer food waste, several limitations of this research warrant acknowledgment, and offer promising avenues for future investigation.

First, our studies are subject to limitations related to sample representativeness and cultural specificity, which constrain the generalizability of our findings. Three of our four studies (Studies 1, 3, and 4) recruited participants from Chinese university student samples, and Study 1 supplemented this sample with adult participants from a Chinese online panel. While student samples are widely used in experimental consumer behavior research for their ability to isolate psychological mechanisms with high internal validity, they differ systematically from the general population in key ways: university students typically have limited and fixed disposable income, different budget management habits, and distinct food consumption patterns compared to household food decision-makers, who are responsible for the majority of household food waste in many markets. Relatedly, our samples exhibit a gender imbalance (approximately 66% female participants across studies), which reflects the demographic structure of our recruitment pools rather than intentional sampling bias. While our robustness analyses confirm that the core effects of price promotions on food waste are consistent across male and female participants in our samples, and that gender does not moderate the focal relationship, the gender imbalance may limit the generalizability of our findings to populations with more balanced gender distributions. In addition, all of our studies were conducted in a Chinese cultural context. China has a unique food culture, dining norms, and a rapidly growing retail and food service market with extremely prevalent promotional activities. Cross-cultural research has documented significant differences in consumer food waste behavior across individualist and collectivist cultural contexts (Porpino et al., 2016), as well as differences in consumers’ responses to price promotions and mental accounting practices. It remains an open question whether the effect of price promotions on food waste, and the mediating role of perceived resources, generalize to Western markets or other cultural contexts. Future research could replicate our findings across more diverse demographic samples (e.g., household grocery shoppers, middle-aged consumers) and cross-cultural settings to test the boundary conditions of our effects across different populations and cultural norms.

Second, our research is limited by the scope of food waste scenarios and food categories examined, which constrains the generalizability of our findings. As acknowledged by prior research, consumer food waste arises from multiple distinct causes, including over-purchasing and stockpiling leading to spoilage, food safety concerns related to expiration dates, and sensory dissatisfaction with food quality (Farr-Wharton et al., 2014; Schanes et al., 2018). All of our experimental studies use a scenario where food waste is triggered by disappointment with the taste of the product (bitter/unpalatable food), which only captures waste driven by quality dissatisfaction. We do not examine waste arising from over-purchasing, which is often cited as the most direct link between price promotions and household food waste (Van Lin et al., 2023). While our core theoretical mechanism (perceived resources) should apply to these other waste contexts—for example, consumers with higher perceived resource abundance may be less likely to plan for and use up stockpiled food, leading to greater spoilage—our studies do not explicitly test this. Future research should extend our model to examine how price promotions influence food waste via both the psychological mechanism identified in this research and the over-purchasing/stockpiling pathway, to provide a more comprehensive understanding of how promotions drive food waste across different consumption stages. In addition, our studies focus on relatively low-value food items (e.g., cookies, pizza, canteen meals). Prior research shows that the value of food is a key predictor of waste behavior, with consumers less likely to waste high-value food items such as meat, seafood, or premium packaged foods (Aschemann-Witzel et al., 2015). It is possible that the pro-waste effect of price promotions is attenuated for high-value foods, as the economic cost of discarding the food may override the effect of enhanced perceived resources. Future research could examine the moderating role of food value and product category in the relationship between price promotions and food waste.

Third, our research has limitations related to the operationalization of price promotions and the measurement of key constructs. In our studies, we operationalize price promotions as direct percentage discounts and fixed-amount coupons, which are common promotional formats but do not capture the full range of promotional tools used in the food industry. For example, buy-one-get-one-free (BOGOF) promotions, bundle discounts, quantity discounts, loyalty program rewards, and free gift promotions are widely used in food retail and catering, and may have different effects on consumers’ perceived resources and subsequent food waste behavior. For instance, quantity discounts may drive both over-purchasing and enhanced perceived resource abundance, leading to a compound effect on food waste. Future research could compare the effects of different promotional formats on consumer food waste, and identify which types of promotions minimize unintended waste while still delivering commercial benefits. In addition, our experimental studies measure food waste intention rather than actual long-term food waste behavior. While meta-analytic evidence confirms that behavioral intention is the strongest proximal predictor of actual behavior (Sheeran, 2002), and our field experiment validates the effect on actual food waste in a dining context, we are unable to capture long-term household food waste behaviors (e.g., spoilage of stockpiled food over time) with our current research design. Future research could use longitudinal designs, household waste diaries, or smart home waste tracking technologies to examine the long-term effects of price promotions on household food waste behavior.

Beyond addressing these limitations, future research can extend our theoretical framework in several meaningful ways. First, future studies could explore additional boundary conditions that moderate the effect of price promotions on food waste, either by shaping consumers’ perceived resources or by altering the cost of food waste. For example, individual difference variables such as price consciousness, deal proneness, environmental concern, and frugality may strengthen or weaken the effects we document. Second, future research could examine whether other elements of the marketing mix (e.g., product packaging, advertising claims, in-store atmospherics) interact with price promotions to influence consumers’ food waste behavior. Third, our research focuses on the individual-level psychological effects of price promotions, but future work could explore the effect of promotions on food waste in social dining contexts, where social norms and interpersonal dynamics play a key role in shaping waste behavior (Qian et al., 2021). Finally, future research could design and test scalable interventions to mitigate promotion-induced food waste, in partnership with retailers, food service operators, and policymakers, to translate our theoretical insights into real-world reductions in consumer food waste.

## Data Availability

The data presented in this study are available on request from the corresponding author.
